# Continuous expression of reprogramming factors induces and maintains mouse pluripotency without specific growth factors and signaling inhibitors

**DOI:** 10.1111/cpr.13090

**Published:** 2021-07-01

**Authors:** Yihuan Mao, Libin Wang, Bei Zhong, Ning Yang, Zhikun Li, Tongtong Cui, Guihai Feng, Wei Li, Ying Zhang, Qi Zhou

**Affiliations:** ^1^ State Key Laboratory of Stem Cell and Reproductive Biology Institute of Zoology Chinese Academy of Sciences Beijing China; ^2^ Institute for Stem Cell and Regenerative Medicine Chinese Academy of Sciences Beijing China; ^3^ University of Chinese Academy of Sciences Beijing China; ^4^ Beijing Institute for Stem Cell and Regenerative Medicine Chinese Academy of Sciences Beijing China; ^5^ College of Life Science Northeast Agricultural University of China Harbin China

**Keywords:** culture medium, gene‐modified animals, germline transmission, pluripotency, reprogramming factors, self‐renewal

## Abstract

**Objectives:**

Derivation and maintenance of pluripotent stem cells (PSCs) generally require optimized and complex culture media, which hinders the derivation of PSCs from various species. Expression of Oct4, Sox2, Klf4, and c‐Myc (OSKM) can reprogram somatic cells into induced PSCs (iPSCs), even for species possessing no optimal culture condition. Herein, we explored whether expression of OSKM could induce and maintain pluripotency without PSC‐specific growth factors and signaling inhibitors.

**Methods:**

The culture medium of Tet‐On‐OSKM/Oct4‐GFP mouse embryonic stem cells (ESCs) was switched from N2B27 with MEK inhibitor, GSK3β inhibitor, and leukemia inhibitory factor (LIF) (2iL) to N2B27 with doxycycline. Tet‐On‐OSKM mouse embryonic fibroblast (MEF) cells were reprogrammed in N2B27 with doxycycline. Cell proliferation was traced. Pluripotency was assessed by expression of ESC marker genes, teratoma, and chimera formation. RNA‐Seq was conducted to analyze gene expression.

**Results:**

Via continuous expression of OSKM, mouse ESCs (OSKM‐ESCs) and the resulting iPSCs (OSKM‐iPSCs) reprogrammed from MEF cells propagated stably, expressed pluripotency marker genes, and formed three germ layers in teratomas. Transcriptional landscapes of OSKM‐iPSCs resembled those of ESCs cultured in 2iL and were more similar to those of ESCs cultured in serum/LIF. Furthermore, OSKM‐iPSCs contributed to germline transmission.

**Conclusions:**

Expression of OSKM could induce and maintain mouse pluripotency without specific culturing factors. Importantly, OSKM‐iPSCs could produce gene‐modified animals through germline transmission, with potential applications in other species.

## Graphical abstract

The requirement of optimized and complex culture media for obtaining and maintaining pluripotent stem cells (PSCs) hinders stem cell research. Here, Mao et al report that continuous expression of reprogramming factors OSKM induces and maintains mouse pluripotency without specific culturing factors. Importantly, the resulting iPSCs can produce gene‐edited animals via germline transmission, possessing a potential application in other species.

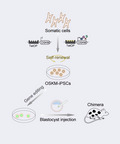

## 1 INTRODUCTION

Pluripotent stem cells (PSCs), including embryonic stem cells (ESCs)^1^, ^2^ and induced pluripotent stem cells (iPSCs),^3^, ^4^, ^5^ have revolutionized research on embryonic development, genome function, and disease modeling. Furthermore, PSCs hold unprecedented potential in regenerative medicine. External signaling pathways^6^ integrate with the internal core transcriptional network to stabilize PSC state.^7^, ^8^ Serum with the added leukemia inhibitory factor (LIF) served as the traditional culture medium for derivation of ESCs from certain mouse strains. Serum supplies the bone morphogenetic protein (BMP), which induces inhibitor‐of‐differentiation proteins to repress differentiation.^9^ BMP can replace serum to maintain mouse ESCs in combination with LIF.^10^ LIF activates STAT3 to inhibit ESC differentiation and promote viability.^11^, ^12^, ^13^ However, these culture conditions have only succeeded in deriving ESCs from certain mouse strains and have failed in other mouse strains and other species. Subsequently, it was supposed that ESCs were in an intrinsic and self‐sufficient cell state once being well protected from differentiation stimuli, including autocrine FGF4 (an activator of the ERK pathway).^14^ Based on this assumption, the MEK/ERK inhibitor PD0325901, GSK3β inhibitor ChIR99021 (2i) condition was established to robustly maintain undifferentiated and homogenous mouse ESCs and derive ground state ESCs from mouse embryos.^14^ The MEK/ERK inhibitor blocked the differentiation of ESCs. GSK3 inhibition resulted in the activation of β‐catenin in canonical WNT pathways, which abrogated the repressive effects of TCF3 on core pluripotency genes including *Esrrb*.^15^, ^16^ Importantly, 2i along with LIF (2iL) overcame the mouse recalcitrant strain barrier and derived ESCs from all mouse strains^17^, ^18^ and even the rat.^19^, ^20^

The core pluripotency regulatory network guaranteed the self‐renewal and pluripotency state. Transcription factors (TFs) OCT4, SOX2, and NANOG cross‐regulate each other and occupy the core of the TF hierarchy that sustains self‐renewal and restricts differentiation of PSCs.^21^ *Oct4* and *Sox2* were reported to be indispensable for mouse ESCs. While *Nanog* ^22^ and *Klf4* ^23^ were individually dispensable, whereas their overexpression could support self‐renewal, respectively. A previous report also claimed that *Myc* could support self‐renewal and pluripotency.^24^ These factors jointly exerted a critical role in reconstructing the genetic regulatory network of ESCs, as was confirmed by the outbreaking finding that Yamanaka factors Oct4, Sox2, Klf4, and c‐Myc (OSKM) were sufficient to reprogram somatic cells into iPSCs under ESC culture conditions, which resets cellular plasticity to a state akin to that of ESCs.^3^

Based on studies in rodents, it has been generally thought that the achievement of pluripotency depends on fine adjustments in the growth factors and signaling inhibitors in the culture media.^6^, ^25^ Nonetheless, the appropriate culture conditions ensuring rodent pluripotency could not be applied to efficiently derive PSCs from other species such as domestic mammals, and the derivation of ESCs from domestic species has undergone a long and unproductive past.^26^

However, more evidences revealed that the evolutionarily conserved TF cocktail OSKM could reprogram somatic cells of non‐rodent species such as the pig,^27^, ^28^ marmoset,^29^ rabbit,^30^ and horse^31^ into putative iPSCs or iPSC‐like cells, under the inappropriate culture conditions “borrowed from” other species such as the human and mouse. These reports demonstrated the importance of reprogramming factors in driving self‐renewal and pluripotency state. Thus, we proposed that reprogramming factors may be able to induce and support PSCs even without the support of specific growth factors and signaling inhibitors. Herein, we explore this possibility by using the classical reprogramming factors OSKM in the mouse. We successfully induced and maintained mouse iPSCs from somatic cells via the continuous expression of OSKM without PSC‐specific growth factors and signaling inhibitors. The resulting iPSCs could contribute to germline transmission, permitting the generation of gene‐edited mice.

## 2 MATERIALS AND METHODS

### 2.1 Animals

The Tet‐On‐OSKM mice were described in previous studies.^32^, ^33^ They carry a doxycycline (DOX)‐inducible reverse tetracycline trans‐activator (M2rtTA) in the *Rosa26* locus, and a single polycistronic OSKM transgene in the *Col1a1* locus. Oct4‐GFP mice carried a GFP under control of the endogenous *Oct4* distal promoter. Tet‐On‐OSKM mice and Oct4‐GFP mice were both obtained from the Jackson Laboratory. The SCID mice used for teratoma formation were purchased from Beijing Vital River Laboratory Animal Technology Co., Ltd. All experiments involving animals were approved by the Institutional Animal Use Committee of the Institute of Zoology, Chinese Academy of Sciences in Beijing.

### 2.2 Embryonic stem cell derivation and culture

The Tet‐On‐OSKM/Oct4‐GFP mouse ESC line was derived from the blastocysts obtained from crossbreeding of the above DOX‐OSKM mice and Oct4‐GFP mice according to standard procedures. The cells were derived and further cultured in 2iL medium on the mitomycin‐c treated mouse embryonic fibroblast (MEF) cells (feeder cells). The detail components of slightly modified N2B27 medium^14^ were listed in Table S1. The 2iL medium^14^ contained N2B27 medium with the addition of PD0325901 (Stemgent, 04‐0006), CHIR99021 (Stemgent, 04‐0004), and LIF (Millipore, ESG1007). ESCs cultured in 2iL medium were switched into three different types of medium: N2B27 with 2i and LIF as the 2iL group, N2B27 with 2 μg/mL DOX (Sigma‐Aldrich) as the OSKM group, and N2B27 as the N2B27 group.

### 2.3 Induced pluripotent stem cell induction and culture

To generate induced pluripotent stem cells (iPSCs), Tet‐On‐OSKM MEFs were seeded onto the feeder cells at a density of 20000 cells per well in 6‐well‐plates. There were two types of induction media. The induction medium of the control group comprised 2iL with DOX. Once iPS‐like clones were picked up to culture in new dishes, DOX was withdrawn. The induction medium of OSKM group consisted of N2B27 with DOX, and the DOX was continued to be supplied throughout daily culture.

### 2.4 Generation of GFP transgenic OSKM‐iPSCs

The PiggyBac (PB) transposon system was used. A PB transposase enzyme (PBase) vector and a PB‐GFP vector were constructed. The PBase vector contained the EF1a promoter and the coding sequence of the PBase. The CAG promoter, 3 × HA, GFP, and polyA sequences were cloned into the PiggyBac backbone to form the PB‐GFP vector (Figure S4A). These two vectors were transfected into OSKM‐iPSCs by using the Neon transfection system (Invitrogen, MPK5000). After 3 days, GFP‐positive cells were sorted via fluorescence‐activated cell sorting (FACS) and were seeded into 6‐well plates. Approximately 6‐8 days later, clones with all cells inside expressing GFP were collected.

### 2.5 Growth curves

To generate growth curves for ESCs and iPSCs, the Cell Counting Kit‐8 (CCK‐8, Sigma‐Aldrich, 96992) was used. After seeding 2500 cells/well in a 48‐well dish, a 1/10 volume of CCK‐8 solution was added to the medium for a two‐hour‐incubation at days 1, 2, 3, 4, and 5. The absorbance of each well at 450 nm was measured using a microplate reader. All the experiments were performed in quadruplicate.

### 2.6 Karyotype analysis

Cells were incubated with 0.05 μg/mL Colcemid for 2‐3 hours. After trypsinization, cells were suspended in 0.075 M KCl at 37°C for 30 minutes. Then, the cells were fixed in solution consisting of methanol and acetic acid (3:1 in volume) for 30 minutes on ice and were dropped onto precooled slides. The cells were stained with Giemsa stain (Sigma‐Aldrich, GS500ML) under standard procedures.

### 2.7 Immunofluorescence staining and alkaline phosphatase staining

Cells were fixed with 4% paraformaldehyde (PFA) for 10 minutes and were subsequently permeabilized and blocked with 0.5% Triton X‐100 (Sigma‐Aldrich) plus 2% BSA (Sigma‐Aldrich, A7906‐100G) for 1 hour. Then, cells were incubated in primary antibody solution overnight at 4℃ and secondary antibodies (donkey anti‐rabbit, Invitrogen, A21206) at room temperature for 1 hour. The primary antibodies were as follows: anti‐OCT4 (Abcam, ab19857), anti‐NANOG (Abcam, ab80892), anti‐SOX2 (Abcam, ab97959), anti‐SSEA1 (Abcam, ab16285), and anti‐TUJ1 (Biolegend, 802001). DNA was stained with Hoechst 33342 (Thermo Fisher Scientific) for 10 minutes. Images were captured using a two‐photon confocal laser scanning microscope (Leica, TCS Sp8). The BCIP/NBT Alkaline Phosphatase Color Development Kit (Beyotime, C3206) was applied to perform alkaline phosphatase staining according to the manufacturer's instructions.

### 2.8 PCR genotyping

KOD One™ PCR Master Mix ‐Blue (TOYOBO, KMM‐201) was used for PCR. The sequences of the primer pair were listed in Table S2. The PCR protocol was: 95℃/1 minutes (1 cycle), 94℃/30 seconds, 70℃/45 seconds (2 cycles), 94℃/30 seconds, 68℃/45 seconds (5 cycles), 94℃/20 seconds, 66℃/1 minutes (29 cycles), and 4℃ hold.

### 2.9 Real‐time quantitative PCR

Total RNA was extracted using the TRIzol reagent (Thermo Fisher Scientific, 15596018). RNA was reverse transcribed using a ReverTra Ace® qPCR RT Master Mix with gDNA Remover Kit (TOYOBO, FSQ‐301). QuantStudio 6 Pro Real‐Time PCR System (Thermo Fisher Scientific) was used to perform the real‐time quantitative PCR analysis with THUNDERBIRD SYBR^®^ qPCR Mix (TOYOBO, QPS‐201) plus 50 × ROX reference dye (TOYOBO, QPS‐201). All these kits were used in accordance with the manufacturer's guidelines. Equal loading was achieved by amplifying GAPDH mRNA. The primers used were listed in Table S3. All reactions were conducted in triplicate.

### 2.10 RNA‐Seq library preparation and data analysis

Total RNA was extracted from cells with the TRIzol reagent (Thermo Fisher Scientific, 15596018). The Illumina platform was applied for RNA‐Seq. Reads were aligned to the mouse reference genome assembly (GRCm38/mm10) using STAR (version 2.7.1a)^34^ with default parameters, and a customized script was used to filter the uniquely mapped reads. The normalized gene expression level (Fragments Per Kilobase Million or FPKM) was obtained using Stringtie (version 2.0),^35^ and the analysis of differentially expressed genes (DEGs) was performed using Cuffdiff (version 2.2.1) with default parameters where two‐fold changes of gene FPKM and *P*‐value < .05 from the Cuffdiff application (http://cole‐trapnell‐lab.github.io/cufflinks/) were regarded as the cutoff values. All‐gene FPKMs were transformed by log2 and used to create scatterplots by R. Gene‐enrichment and functional annotation analysis was performed using the David tool.^36^ Heatmaps were generated using log‐transformed gene FPKM with the R “pheatmap” function (https://cran.r‐project.org/web/packages/pheatmap/index.html). After the log transformation, gene FPKM values (larger than 1 in at least one sample) were used in principal component analysis (PCA) with the R “prcomp” function and in the hierarchical clustering analysis with the R “hclust” function. Published RNA‐Seq data were downloaded from NCBI GEO GSE97954,^37^ and the R “sva” function was used to eliminate the batch effect.^38^

### 2.11 Teratoma formation, embryoid body formation, and histological analysis

Cells were trypsinized and collected, and cell suspensions were injected subcutaneously into the flanks of SCID mice. Approximately 1 × 10^7^ cells were used in each injection. Teratomas were excised about one month later and were fixed and stained with hematoxylin and eosin (H&E) to perform histological analysis. For embryoid body (EB) formation, cells were harvested by trypsinization and seeded into bacterial culture dishes in the N2B27 medium.

### 2.12 Blastocyst injection

Blastocyst injection was performed according to the procedures given in a previous report.^39^ Briefly, diploid blastocysts were collected from the uterus of 3.5 days post coitum (dpc) super‐ovulated female CD‐1 mice after mating with male CD‐1 mice. Cells were harvested by trypsinization and 12‐15 cells were microinjected into each blastocyst. After 1‐4 hours of culture, these processed embryos were transferred into the oviduct of pseudo‐pregnant CD‐1 mice at 0.5 dpc. Chimeras were identified by GFP expression or coat colors.

### 2.13 Statistical analysis

For statistical analysis, Students’ *t* test was used in GraphPad Prism 8 software. In all figures: *P value < .05; **P value < .01; ***P value < .001; ****P value < .0001.

## 3 RESULTS

### 3.1 Induced expression of OSKM maintained self‐renewal and pluripotency of mouse ESCs after withdrawal of 2i and LIF

**FIGURE 1:**
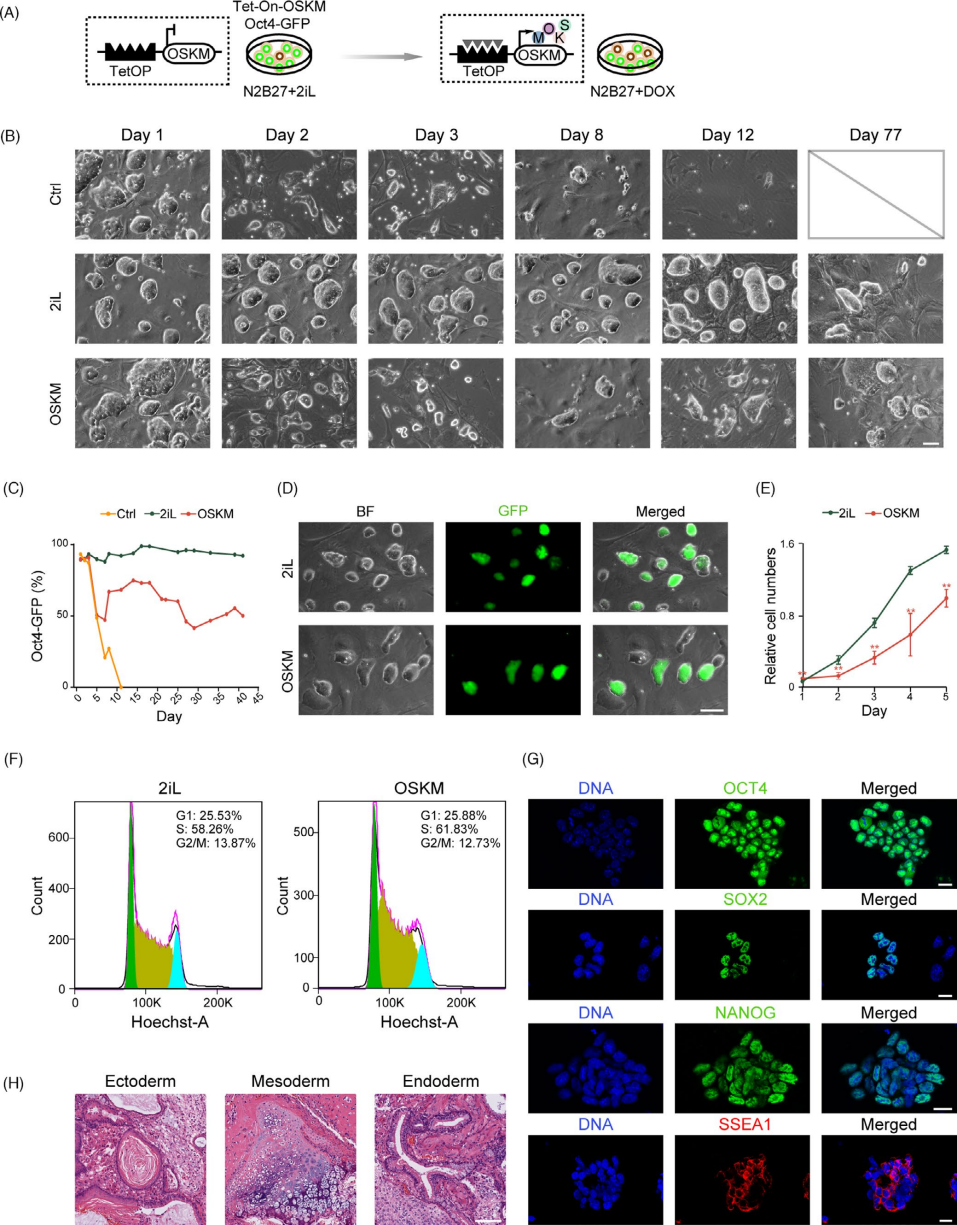
Mouse ESCs maintained by induced expression of OSKM upon withdrawal of 2iL. A, Schematic of pluripotency maintenance in mouse ESCs via expression of OSKM without 2iL. The Tet‐On‐OSKM/Oct4‐GFP ESC line harbored a DOX (Doxycycline)‐induced single‐copy OSKM (*Oct4*, *Sox2*, *Klf4*, and *c‐Myc*) transgenic cassette and a GFP reporter driven by endogenous *Oct4* distal promoter. The culture medium was switched from N2B27 with 2iL to N2B27 with 2 μg/mL DOX. B, Colony morphology of ESCs cultured under different conditions at selected time points. Ctrl (control), N2B27 group; 2iL, N2B27 with 2iL group; and OSKM, N2B27 with DOX group. Scale bar, 75 μm. C, The percentages of GFP‐positive cells of ESCs cultured under three different conditions at selective time points. D, Morphology of ESCs cultured under 2iL and OSKM conditions, respectively. GFP‐positive cells could be observed. Scale bar, 50 μm. E, Statistical analysis of the relative cell numbers of ESCs cultured in OSKM medium (OSKM‐ESCs) and ESCs cultured in 2iL medium (2iL‐ESCs) at indicated time points. Cell Counting Kit‐8 (CCK‐8) was used for data collecting. Data were represented as mean ± SEM. **P < .01. F, FACS analysis of DNA content of ESCs under 2iL and OSKM conditions. Percentages of cells in G1, S, and G2/M phases were shown. G, Immunostaining for ESC markers OCT4, SOX2, NANOG, and SSEA1 of OSKM‐ESCs. DNA was stained with Hoechst 33342. Scale bars, 20 μm. H, Histological section analysis of the teratomas derived from OSKM‐ESCs showed differentiation into all three germ layers (ectoderm, mesoderm, and endoderm). Scale bar, 200 μm

To test whether continuous OSKM expression could maintain the cell identity of mouse ESCs, we used an ESC line stably expressing the pluripotency reporter Oct4‐DE‐GFP (GFP under control of the endogenous Oct4 distal promoter) and the single polycistronic OSKM transgene upon the addition of DOX (Figure 1A). This cell line was derived and cultured in N2B27 medium supplemented with the MEK inhibitor PD0325901, GSK3 inhibitor ChIR99021, and LIF (2iL medium). Next, the culture medium was switched from 2iL to N2B27 with DOX (OSKM medium) and the OSKM transgene was continuously activated (Figure 1A), whereas N2B27 medium alone was used as the negative control condition, and 2iL medium acted as the positive control condition. The ESCs in the 2iL medium (hereinafter referred to 2iL‐ESCs) maintained their typical ESC morphology and >90% of the cell population was Oct4‐GFP positive (Figure 1B‐D). However, ESCs cultured in N2B27 medium rapidly underwent cell differentiation and high rates of death, barely surviving after day 11 (Figure 1B,C). Nevertheless, ESCs cultured in OSKM medium (OSKM‐ESCs) survived and finally maintained typical ESC morphology for more than 77 days (38 passages) and 40%‐60% of cells were Oct4‐GFP positive (Figure 1B‐D). Different dosages of DOX exerted different effects on cell survival and generated different proportions of Oct4‐GFP, where 2 μg/mL was determined as the optimal concentration (Figure S1A). ESCs cultured in OSKM medium continued to proliferate for over 38 passages, although the growth rate was slower than that of 2iL‐ESCs (Figure 1B,E).

In summary, these results demonstrated that OSKM expression maintained self‐renewal of mouse ESCs after withdrawal of 2iL. The cell cycle analysis indicated that OSKM‐ESCs and 2iL‐ESCs shared similar distributions of G1, S, and G2/M phases (Figure 1F). Next, we tested whether OSKM‐ESCs could maintain pluripotency. The cells were alkaline phosphatase (AP) positive (Figure S1B) and expressed typical ESC markers OCT4, SOX2, NANOG, and SSEA1 by immunofluorescent staining (Figure 1G). Importantly, OSKM‐ESCs formed differentiated teratomas with structures of all three germ layers (Figure 1H). Thus, induced continuous expression of OSKM could maintain self‐renewal and pluripotency of mouse ESCs without the support of PSC‐specific culturing factors.

### 3.2 Induction of pluripotency from somatic cells without specific growth factors and signaling inhibitors

**FIGURE 2:**
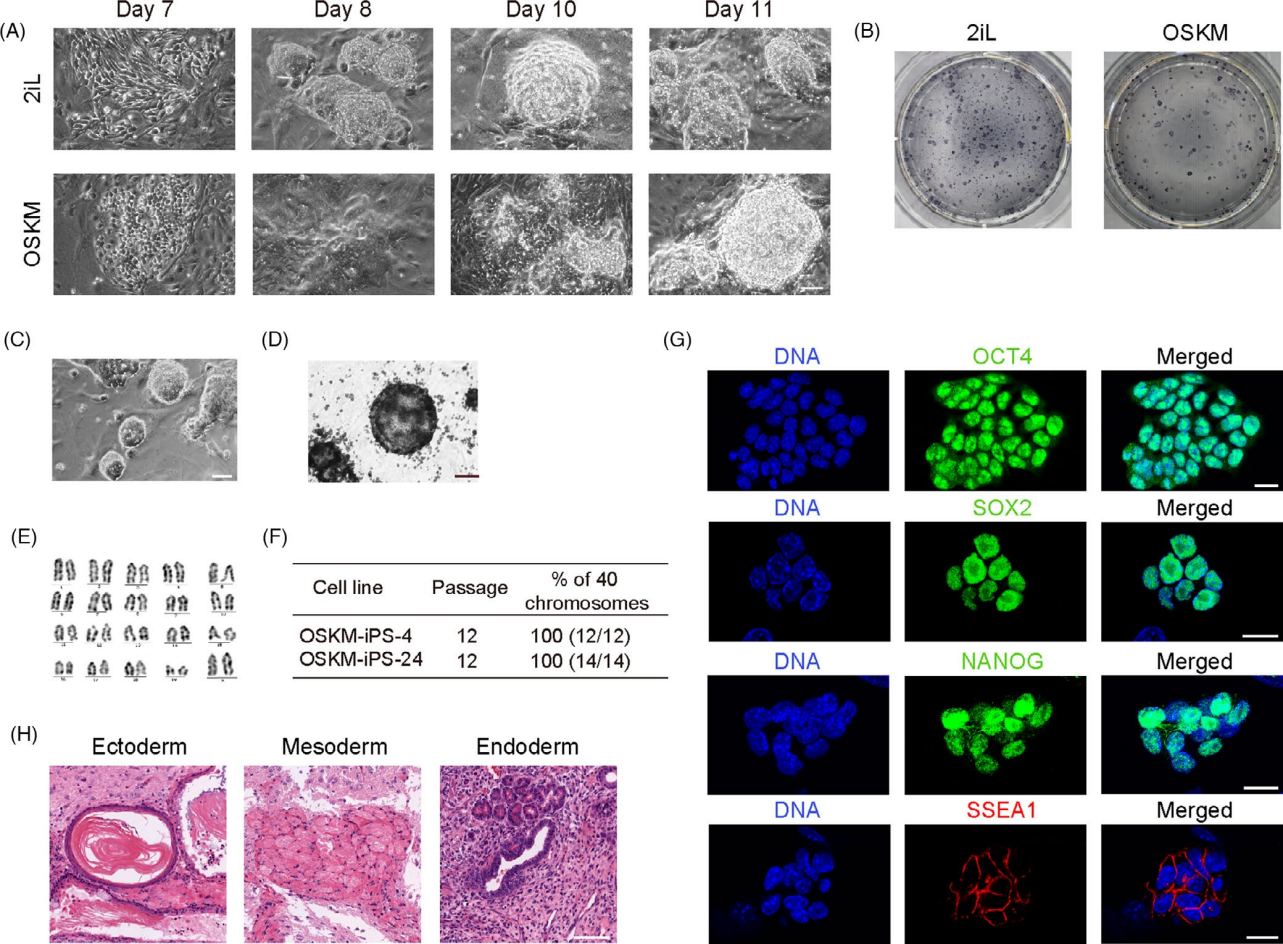
Induction and maintenance of pluripotency via induced OSKM expression. A, Colony morphology of reprogrammed cells in different inductive media at selected time points. Groups of 2iL and OSKM were shown. Scale bar, 75 μm. B, Colonies observed after reprogramming under inductive media of 2iL and OSKM, respectively. Cells were stained by alkaline phosphatase (AP). C, Morphology of stable cell lines of OSKM‐iPSCs at Passage 23. Scale bar, 75 μm. D, AP staining of OSKM‐iPSCs. Scale bar, 75 μm. E, Karyotype analysis of OSKM‐iPS‐4 cell line. F, Statistical graph of karyological characteristics of the two OSKM‐iPSC lines (OSKM‐iPS‐4 and OSKM‐iPS‐24) at Passage 12. G, Immunostaining for ESC markers OCT4, SOX2, NANOG, and SSEA1 of OSKM‐iPSCs. DNA was stained with Hoechst 33342. Scale bars, 20 μm. H, Histological section analysis of the teratomas derived from OSKM‐iPSCs. Ectoderm, mesoderm, and endoderm structures were shown. Scale bar, 200 μm

Next, we determined whether this system could reconstruct pluripotency de novo from MEF cells obtained from the above Tet‐On‐OSKM mice. MEF cells were reprogrammed by OSKM medium (N2B27 supplemented with DOX) and 2iL (N2B27 supplemented with 2iL and DOX, and DOX was withdrawn after reprogramming), respectively. After 14‐18 days of reprogramming (Figure 2A), AP‐positive clones were observed in both groups (Figure 2B,D and Figure S2A). Several clones were randomly selected from the OSKM group. The resulting cell lines could proliferate without visible differentiation beyond Passage 23 (Figure 2C). Cell lines carried normal karyotypes of 40 chromosomes (Figure 2E,F, and Figure S2B). ESC markers, such as OCT4, SOX2, NANOG, and SSEA1 were positive in OSKM‐iPSCs (Figure 2G). They successfully differentiated into structures of all three germ layers, including ectoderm (Figure 2H and Figure S2C,D), mesoderm (Figure 2H), and endoderm (Figure 2H).

### 3.3 Gene expression profiles of OSKM‐ESCs and OSKM‐iPSCs

**FIGURE 3:**
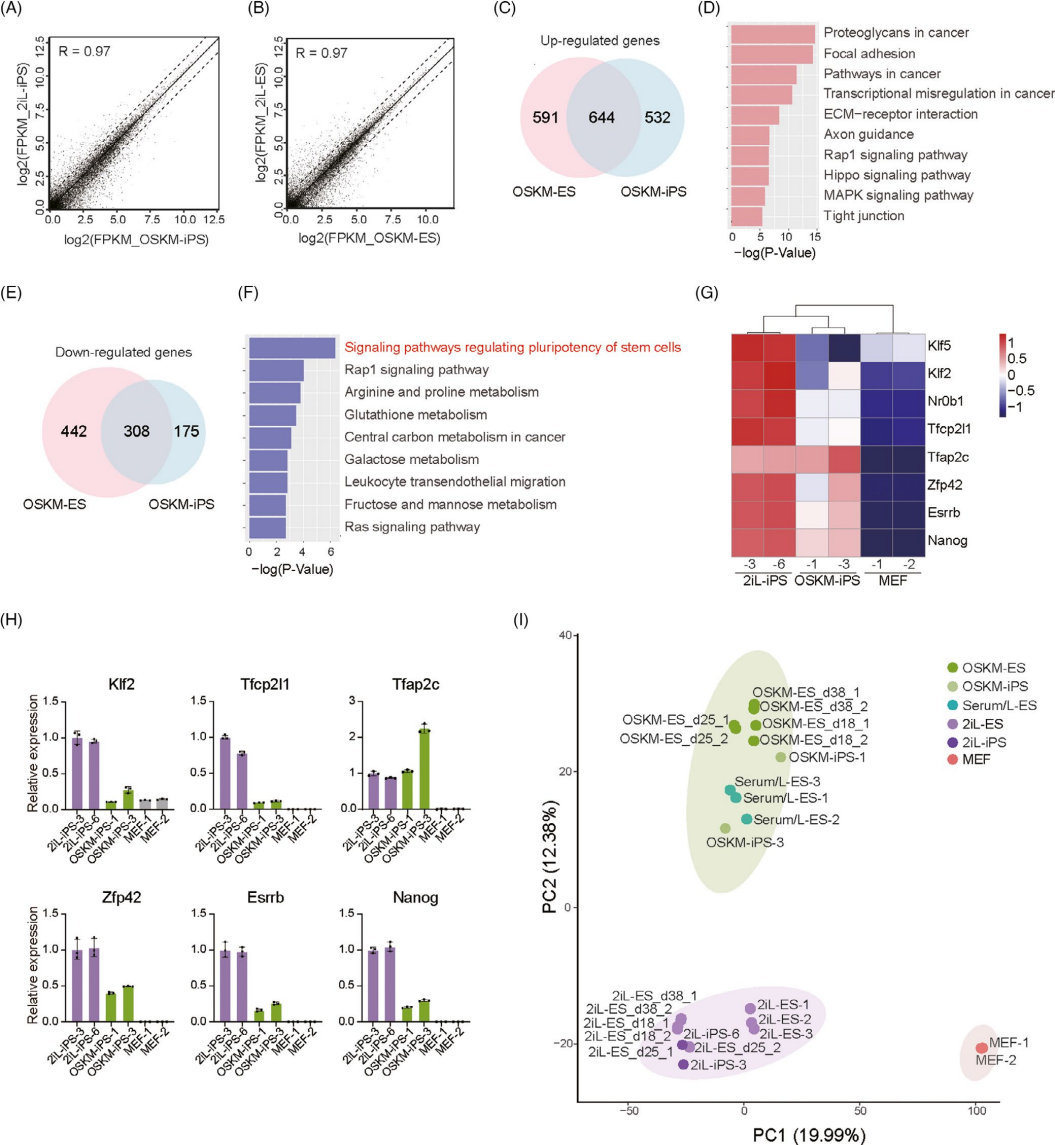
Gene expression patterns of OSKM‐iPSCs and OSKM‐ESCs. A, Gene expression comparison of OSKM‐iPSCs and 2iL‐iPSCs shown in the scatterplot; n = 2. The correlation coefficient (R) was determined by Pearson's correlation. B, Gene expression comparison of OSKM‐ESCs (day 38) and 2iL‐ESCs (day 38) shown in the scatterplot; n = 2. Correlation coefficient (R) was determined by Pearson's correlation. C, Venn diagram showing the numbers of upregulated genes in OSKM‐ESCs when compared to 2iL‐ESCs (left) and in OSKM‐iPSCs when compared to 2iL‐iPSCs (right). Fold change >2, *P* < .05; 644 genes were commonly upregulated in both comparisons. D, Enriched KEGG pathways of above 644 commonly up‐regulated genes. E, Venn diagram showing the numbers of downregulated genes in OSKM‐ESCs when compared to 2iL‐ESCs (left) and in OSKM‐iPSCs when compared to 2iL‐iPSCs (right), fold change >2, *P* < .05; 308 genes were commonly downregulated in both comparisons. F, Enriched KEGG pathways of above 308 commonly downregulated genes. G, Heatmap of expression levels of naïve pluripotency genes in 2iL‐iPSCs, OSKM‐iPSCs, and MEF cells. H, Relative expression levels of naïve pluripotency genes measured by real‐time quantitative PCR in 2iL‐iPSCs, OSKM‐iPSCs, and MEF cells. I, Principal‐component analysis for gene expression of all related samples. PC1 and PC2 represented the top two principal components. Serum/L represented serum with LIF

We performed RNA‐Seq to further characterize the OSKM‐ESCs and OSKM‐iPSCs. Global gene expression profiles of OSKM‐iPSCs were quite similar to 2iL‐iPSCs (Figure 3A). And likewise, gene expression profiles of OSKM‐ESCs were similar to those of 2iL‐ESCs (Figure 3B). Thus, the global gene expression profiles of OSKM PSCs were similar to 2iL PSCs. Next, the DEGs were analyzed. In total, 644 upregulated genes overlapped in both the OSKM‐ESCs versus 2iL‐ESCs and OSKM‐iPSCs versus 2iL‐iPSCs comparisons (1,235 upregulated genes in OSKM‐ESCs and 1176 ones in OSKM‐iPSCs) (Figure 3C). These DEGs were enriched in pathways such as pathway of proteoglycans in cancer and pathway of focal adhesion (Figure 3D). Conversely, 308 DEGs were downregulated both in OSKM‐ESCs and OSKM‐iPSCs (Figure 3E), and they were enriched in signaling pathways regulating the pluripotency of stem cells (Figure 3F). Thus, we compared the expression levels of naïve pluripotency genes of OSKM‐ESCs and OSKM‐iPSCs to those of 2iL‐ESCs and 2iL‐iPSCs. As shown in the heatmap, naïve pluripotency genes, such as *Klf2*, *Esrrb*, *Nanog*, and *Rex1* were consistently expressed to a lower degree in OSKM‐iPSCs compared to 2iL‐iPSCs, while expression levels were higher than those in somatic cells (Figure 3G). Real‐time quantitative PCR confirmed these results (Figure 3H), which were also observed in OSKM‐ESCs when compared to those of 2iL‐ESCs (Figure S3A). The RNA levels of *Sox2* and *Klf4* were comparable to those of 2iL‐iPSCs, although *Oct4* expression was slightly lower than that in 2iL‐iPSCs; and endogenous *Oct4*, *Sox2*, and *Klf4* were activated, albeit endogenous *Klf4* to a lower degree (Figure S3B).

The PCA analysis and hierarchical clustering analysis (Figure 3I and Figure S3C) revealed that both OSKM‐ESCs and OSKM‐iPSCs were clearly distinguished from MEF cells. They were similar in profile in 2iL‐ESCs and 2iL‐iPSCs, and closer to serum/L‐ESCs (ESCs cultured under serum plus LIF). Furthermore, the OSKM‐ESCs collected at different culture timepoints (days 18, 25, and 38) exhibited similar gene expression profiles, indicating the system could maintain cell states stably throughout culture. In conclusion, OSKM sustained an alternative pluripotency state.

### 3.4 Application of OSKM‐iPSCs in producing gene‐edited animals

**FIGURE 4:**
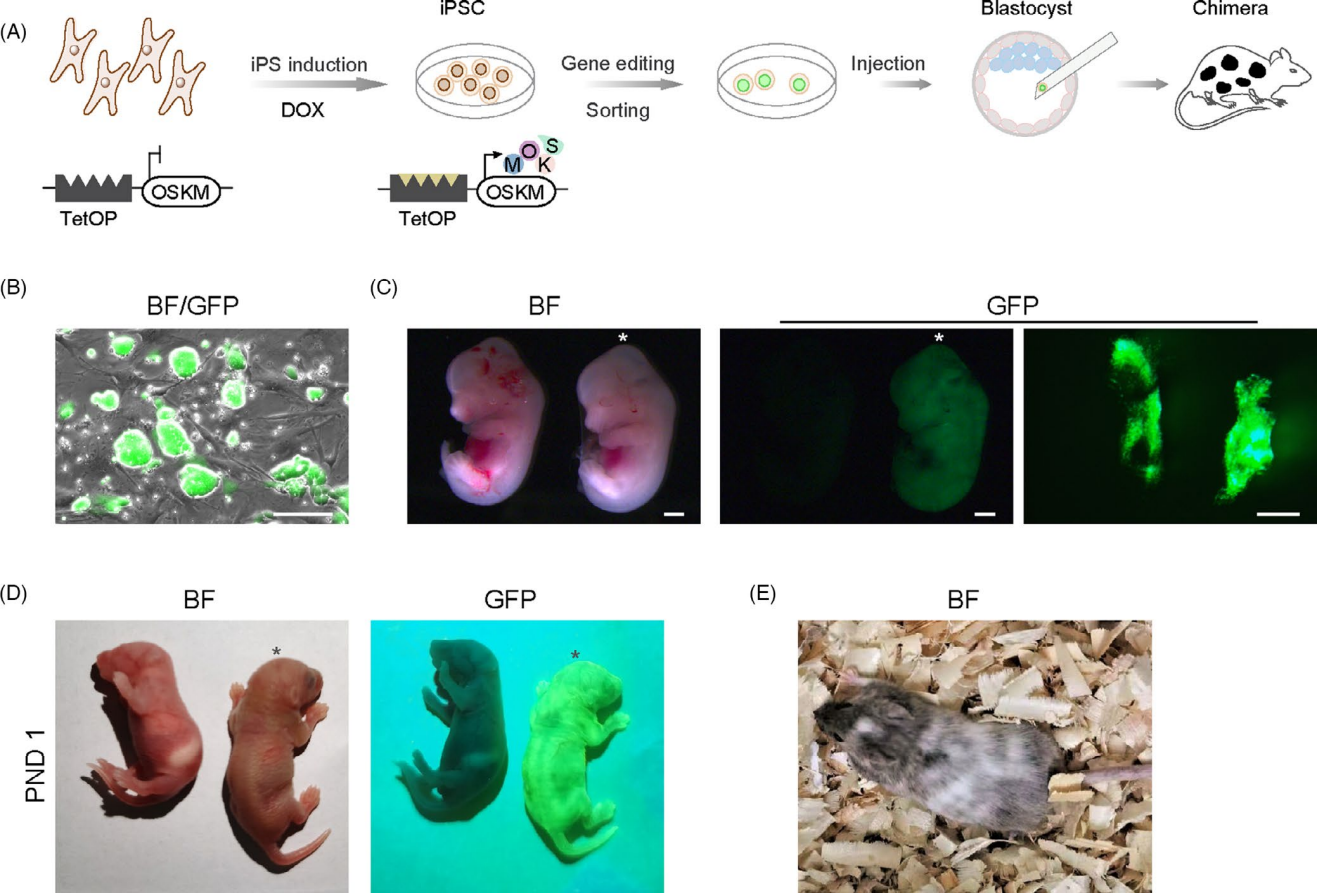
Potential of obtaining gene‐edited animals using OSKM‐iPSCs. A, Schematic of process for obtaining gene‐edited animals using OSKM‐iPSCs. B, Morphology of GFP‐transgenic OSKM‐iPSCs. Scale bar, 50 μm. C, Images of chimeric mouse embryo (days post coitum 12.5) with contribution of OSKM‐iPSCs generated by blastocyst injection (marked by an asterisk). The other served as the negative control. Genital ridges of the chimeric embryo were shown on the right. Scale bars, 500 μm. D, Chimeric mouse at postnatal day 1 (PND 1) with contribution of OSKM‐iPSCs (marked by an asterisk). The other served as the negative control. (E) Adult chimeric mouse with contribution of OSKM‐iPSCs

One major application of PSCs is to produce gene‐edited animal models for genome function research, disease modeling and drug screening. For this purpose, we developed a route to produce chimera mice with germline transmission using transgenic OSKM‐iPSCs (Figure 4A). We randomly selected two OSKM‐iPSC lines and inserted the GFP transgene into their genome using the PiggyBac transposon vector (Figure S4A) to examine their ability to produce chimeras. The resulting GFP‐transgenic subclones were collected (Figure 4B) and were further injected into mouse blastocysts to form chimeras. We dissected embryos at 12.5 days post coitum (dpc), and a chimera embryo with germline chimerism was observed (Figure 4C). Genotyping PCR results in further showed that OSKM‐iPSCs contributed to various organs and tissues (Figure S4B). Chimerism in the newborn and adult mouse was also observed, and genotyping PCR results confirmed the contribution of transgenic OSKM‐iPSCs (Figure 4D,E, and Figure S4C).

## 4 DISCUSSION

Originally, self‐renewal and pluripotency were thought to be supported by interaction between external signaling pathways and intracellular core pluripotency transcription regulatory networks. Obtaining authentic pluripotency was generally considered to depend on fine adjustments in the growth factors and signaling inhibitors supplied in the culture media. In this study, we demonstrated that the simple continuous expression of Yamanaka factors OSKM could induce and maintain self‐renewal and pluripotency without traditional additives. The resulting OSKM‐iPSCs obtained the abilities of long‐term self‐renewal, differentiation into three germ layers in teratoma, and incorporation into the developing embryo with germline‐competence upon blastocyst injection. Although the crucial growth factors and signaling inhibitors can promote maintenance of PSCs and reprogramming process,^40^ our results showed that they might not be indispensable for authentic pluripotency, at least to a certain extent.

**FIGURE 5:**
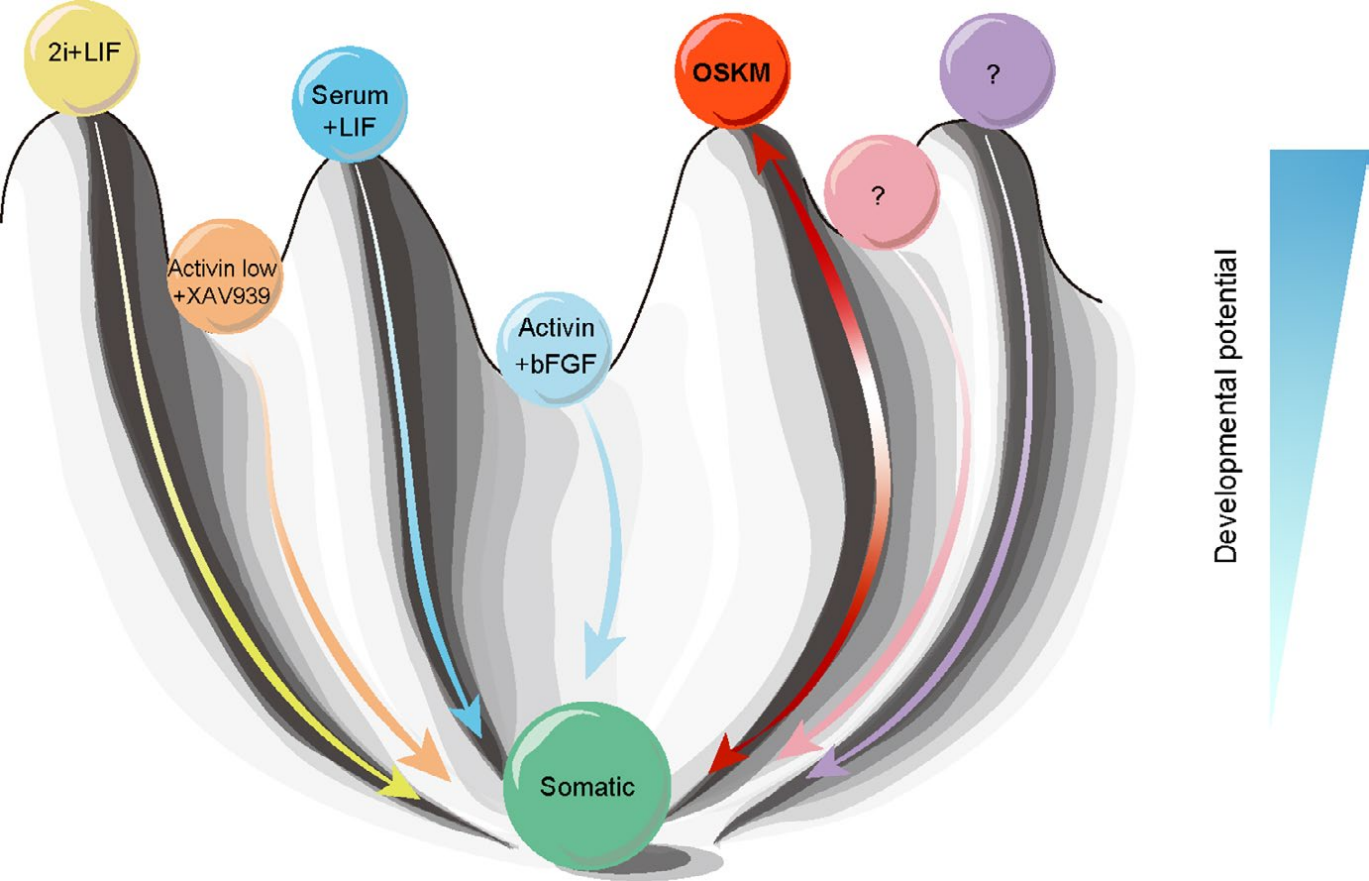
Schematic of several pluripotency states established in mouse. An alternative pluripotency state was established in the OSKM system of this study. This schematic was adapted from Conrad Waddington's model. On the top of the “developmental potential” mountain, the 2iL maintained the cells in a naïve pluripotency state, serum with LIF supported another metastable pluripotency state, activin A (low) with XAV939 (A_lo_XR)^41^ maintained formative pluripotency state, and activin A with bFGF supported the primed pluripotency state.^42^ An alternative pluripotency state was established by continuous expression of OSKM, while bypassing the requirement of specific growth factors and signaling inhibitors. All these PSCs in different states could differentiate into somatic cells. Somatic cells could be reprogrammed into PSCs in our proposed OSKM system

To date, several different pluripotency states of mouse PSCs have been established (Figure 5). On the top of the “developmental potential” mountain, the 2iL culture maintained the cells in naïve pluripotency state, serum with LIF supported another metastable pluripotency state, activin A (low) with XAV939 (A_lo_XR)^41^ maintained the formative pluripotency state, and activin A with bFGF supported the primed pluripotency state.^42^ Here, we established an alternative pluripotency state (Figure 5). The PSCs established in our study were pluripotent, as confirmed by germline transmission, and they showed transcriptional landscapes similar to PSCs cultured in both serum/LIF and 2iL, and more similar to serum/LIF. The expression levels of naïve pluripotency genes were lower in our OSKM‐PSCs than those in PSCs cultured in 2iL condition, which implied that the gene regulatory networks might be reconfigured in the OSKM‐PSCs. A deeper understanding of the gene regulatory networks in OSKM‐PSCs still requires further research. These OSKM‐PSCs we obtained may serve as a useful model to study how these four TFs drive reprogramming and support PSCs in the absence of “essential” PSC‐specific culturing factors. It would also be of interest to determine how the expression of these TFs maintained authentic pluripotency with the naïve pluripotency genes consistently downregulated, which were thought to be positively associated with developmental potential.

As previously reported, the stoichiometry of reprogramming factors applied during reprogramming significantly influenced the resulting pluripotency of iPSCs.^43^ Our present system harbors a single copy transgene of OSKM. In future studies, the comparison among the cell states obtained via different gene combinations and dosages of TF cocktails in parallel can help us to further understand the regulatory effects of TFs on pluripotency. Furthermore, considering the potential risk of tumor formation upon c‐Myc activation,^44^ although the Tet‐On‐OSKM mice did not develop tumors as reported,^43^ we aim to attempt other combinations, bypassing c‐Myc altogether.^45^, ^46^

Due to differences in transcriptional regulatory networks and signaling stimuli among species, and other unclear reasons,^26^, ^47^ the appropriate culture conditions ensuring rodent pluripotency could not be used to efficiently derive authentic PSCs from other species such as valuable domestic mammals. Numerous efforts have been made to optimize culture media in order to obtain authentic PSCs from livestock species, as summarized in several reviews.^26^, ^48^ However, to date, there are no repeatable and reliable culture conditions to derive authentic pluripotency of domestic animals. This largely hinders the advancement of producing gene‐edited animals. Based on our study, we hypothesized that although differences exist among various animal species, there might be a possibility of conserved reprogramming factors establishing a so‐called “common” expandable and pluripotency state across different species. A similar method may be used to create PSCs of other species while bypassing the requirement of complex and specific culture conditions, and to produce gene‐edited animal models. This route might serve as a so‐called “universal” approach to obtain gene‐edited animal models from various species, which requires further study.

## CONFLICT OF INTEREST

The Editor‐in‐Chief of the journal, Professor Qi Zhou, is a co‐author of this article. The Editor‐in‐Chief was blinded to the peer review process. An Associate Editor handled the peer review process for this article and made the final decision as to its suitability for publication.

## AUTHOR CONTRIBUTIONS

Yihuan Mao: investigation, methodology, formal analysis, resources, validation, visualization and writing—original draft; Libin Wang: investigation, methodology, formal analysis, resources, validation and writing—original draft; Bei Zhong: investigation, formal analysis, resources and visualization; Ning Yang: formal analysis, software and visualization; Zhikun Li: investigation and writing—review and editing; Tongtong Cui: formal analysis and writing—review and editing; Guihai Feng: formal analysis and writing—review and editing; Wei Li: funding acquisition and supervision; Ying Zhang: conceptualization, supervision and writing—review and editing; Qi Zhou: conceptualization, funding acquisition, supervision and writing—review and editing.

## Supporting information

Supplementary Material

## Data Availability

The accession number for the RNA‐Seq data reported in this paper is GEO: GSE173471.
