# Supplementary material for: Continuous expression of reprogramming factors induces and maintains mouse pluripotency without specific growth factors and signaling inhibitors
Source: Cell Prolif. 2021 Jul 1;54(8):e13090. doi: 10.1111/cpr.13090 (PMC8349648; doi:10.1111/cpr.13090)
Supplement: Supplementary file 1 — Supplementary Material [file CPR-54-e13090-s001.docx]

# Supplementary information


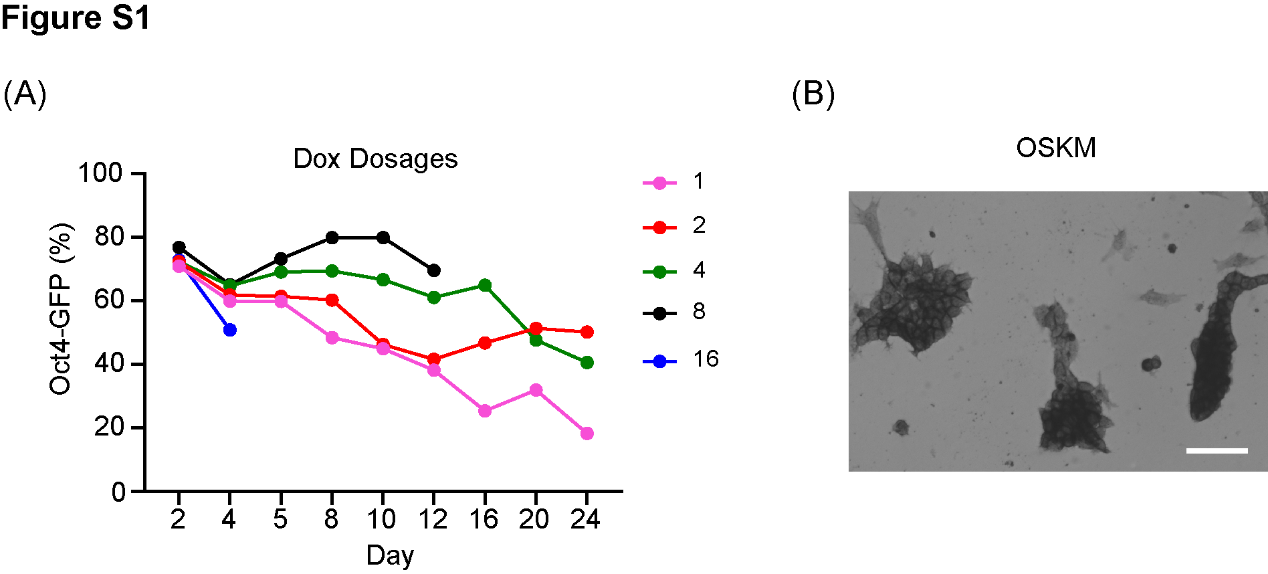


**Figure S1. Mouse ESCs cultured in OSKM condition without 2iL, Related to Figure 1.** (A) The percentages of GFP-positive cells of Oct4-GFP OSKM-ESCs cultured under different DOX dosages (1, 2, 4, 8, and 16 μg/mL) at various time points were shown in the graph. (B) AP staining of OSKM-ESCs. Scale bar, 75 μm.


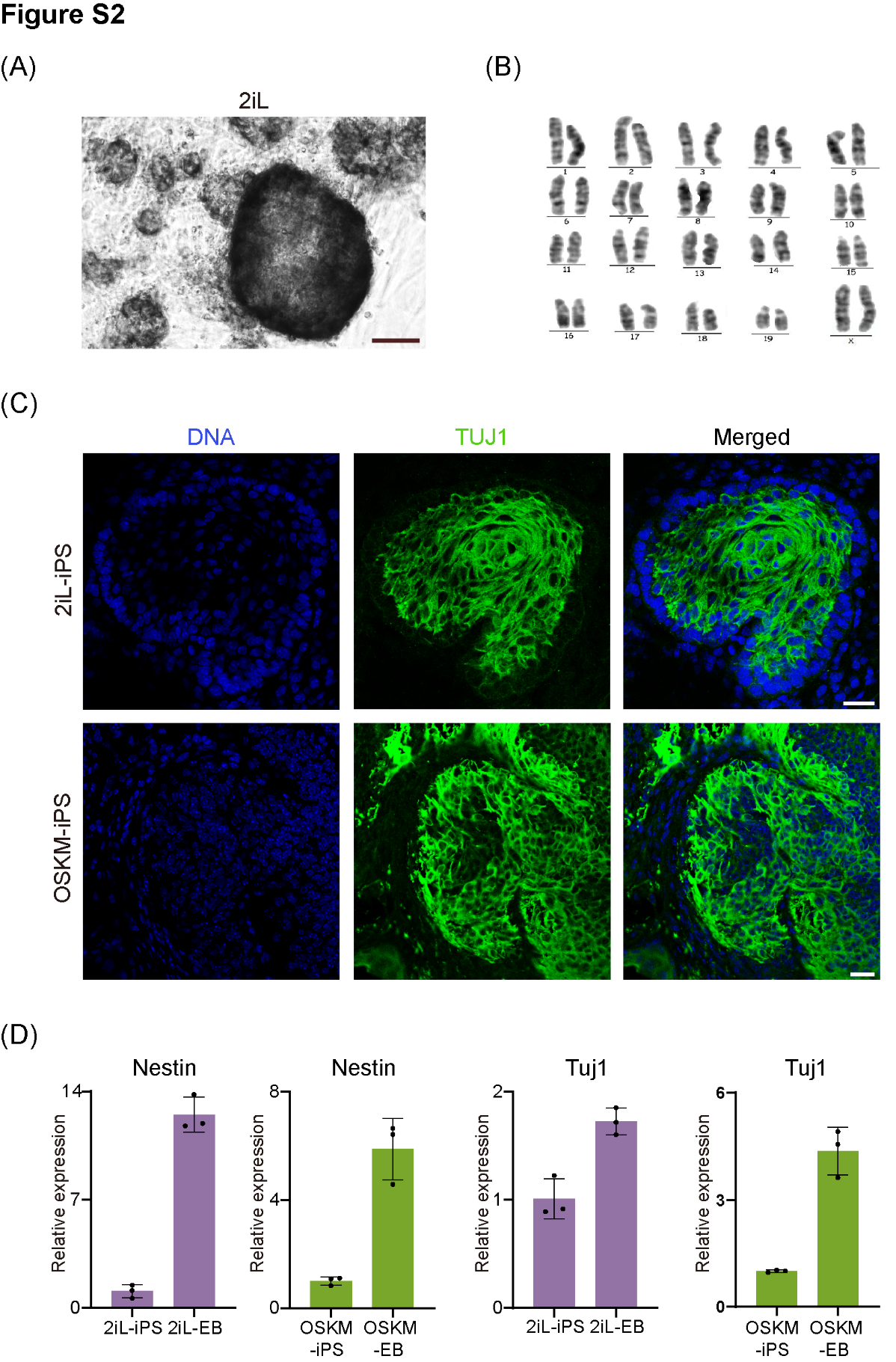


**Figure S2. Induction and maintenance of iPSCs via OSKM expression, Related to Figure 2.** (A) AP staining of 2iL-iPSCs. Scale bar, 75 μm. (B) Karyotype analysis of OSKM-iPS-24 cell line. (C) Immunostaining for neural marker TUJ1 of teratomas derived from OSKM-iPSCs and 2iL-iPSCs, respectively. DNA was stained with Hoechst 33342. Scale bar, 25 μm. (D) Relative expression levels of neural marker genes measured by realtime quantitative PCR in embryoid body cells (day 12) derived from 2iL-iPSCs and OSKM-iPSCs. 2iL-EB represented embryoid body cells derived from 2iL-iPSCs, and OSKM-EB represented embryoid body cells derived from OSKM-iPSCs.


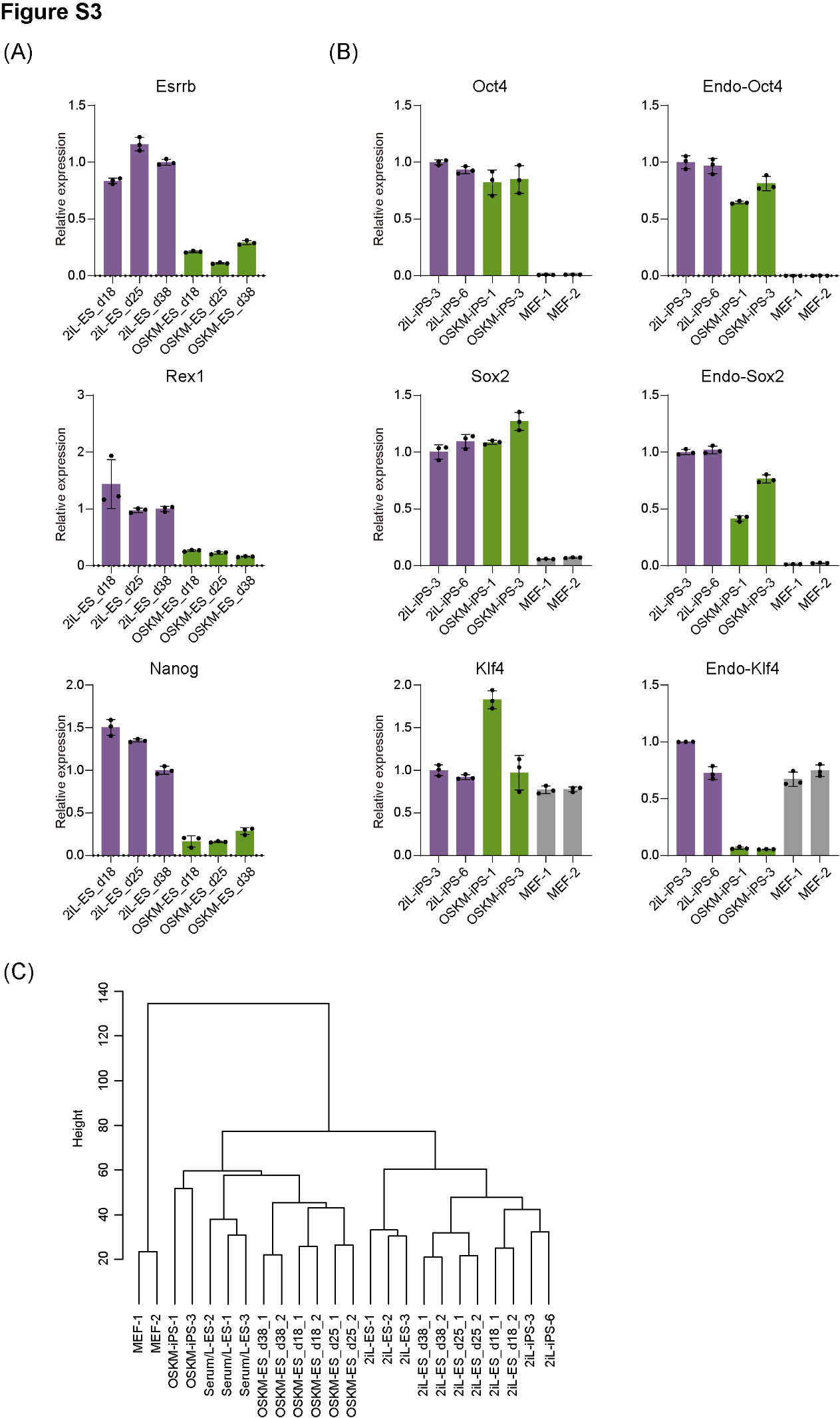


**Figure S3. Gene expression profiles of OSKM-PSCs, Related to Figure 3.** (A) Realtime quantitative PCR analysis of expression levels of *Esrrb*, *Rex1* and *Nanog* in 2iL-ESCs and OSKM-ESCs collected at day 18, 25, and 38; *n* = 3. (B) Realtime quantitative PCR analysis of expression levels of *Oct4*, *Sox2*, *Klf4*, endogenous *Oct4*, endogenous *Sox2* and endogenous *Klf4* in OSKM-iPSCs and MEF cells; Endo represented endogenous; *n* = 3. (C) Hierarchical clustering analysis of the gene expression of all related samples. Y axis represented the Euclidean distances among all samples.


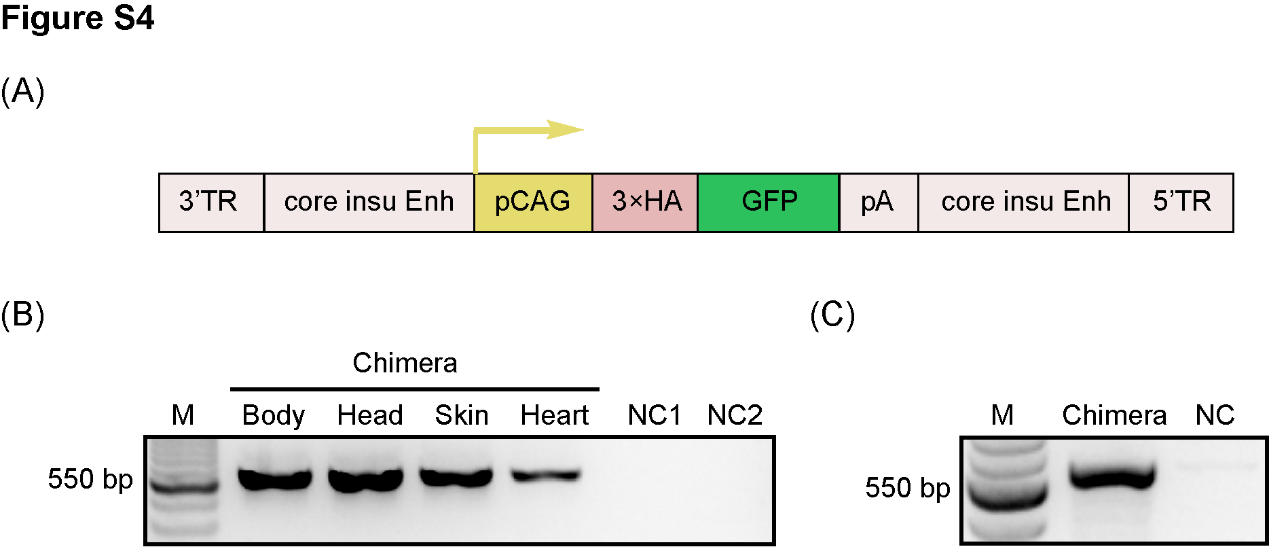


**Figure S4**. **Incorporation of OSKM-iPSCs into embryo with germline-competence, Related to Figure 4.** (A) Schematic of plasmid PB-CAG-GFP; core insu Enh represented core insulation enhancer, pCAG meant CAG promoter, pA represented polyA. (B) Genotyping PCR of different tissues of chimeric embryo in Figure 4C. The body, head, skin, and heart tissues were tested. NC meant negative control. (C) Genotyping PCR of tail tips in chimera mouse in Figure 4E. NC meant negative control.

**Supplementary Tables**

**Table S1. Components of N2B27 medium.**

| **Component** | **Brand, Code** | **Volume** |
| --- | --- | --- |
| DMEM/F12 | Gibco, 12400-024 | 229.2 mL |
| Neurobasal | Gibco, 21103-049 | 229.2 mL |
| Knockout Serum Replacement (KOSR) | Gibco, 10828028 | 25 mL |
| N2 | Gibco, 17502-048 | 2.5 mL |
| B27 | Gibco, 17504-044 | 5 mL |
| GlutaMAX | Gibco, 35050-079 | 5 mL |
| β-Mercaptoethanol | Gibco, 21985 | 911 μL |
| 2% Bovine Serum Albumin (BSA) | Sigma-Aldrich, A7906-100G | 625 μL |
| 10 mg/mL Insulin | Roche applied science, 11376497001 | 500 μL |
| Penicillin-Streptomycin | Gibco, 15140-122 | 5 mL |
| **Total** |  | **500 mL** |

**Table S2. Primers for PCR genotyping.**

| **Primer** | **Sequence (5’ to 3’)** |
| --- | --- |
| F | TTGCTCAGCGGTGCTGTCCA |
| R | GCACAGCATTGCGGACATG |

**Table S3. Primers for realtime quantitative PCR.**

| **Primer** | **Sequence (5’ to 3’)** |
| --- | --- |
| mGAPDH-qF | AGGTCGGTGTGAACGGATTTG |
| mGAPDH-qR | TGTAGACCATGTAGTTGAGGTCA |
| Endo-Oct4-qF | ACTGAGGAGGGATTAAAAGCACAAC |
| Endo-Oct4-qR | GCTATCTACTGTGTGTCCCAGTCTT |
| Endo-Sox2-qF | AAATTAACGCAAAAACCGTGATGCC |
| Endo-Sox2-qR | ATAGTCCCCCAAAAAGAAGTCCCAA |
| Endo-Klf4-qF | TCGGACCTACTTATCTGCCTTGC |
| Endo-Klf4-qR | TCAAACCAAAACCCCCAGATTGC |
| mOct4-qF | GCAGATCACTCACATCGCCAAT |
| mOct4-qR | CCTGGGAAAGGTGTCCCTGTAG |
| mNanog-qF | AGGATGAAGTGCAAGCGGTG |
| mNanog-qR | TGCTGAGCCCTTCTGAATCAG |
| mRex1-qF | GAAGAGAGAGGTCACGCAAGAGACG |
| mRex1-qR | TCGATAAGACACCACAGTACACACCG |
| mEsrrb-qF | CTGCAGCTGGTGCGCAGGTA |
| mEsrrb-qR | CCTGGAGCTTCTGCACCGCC |
| mSox2-qF | ACAGATGCAACCGATGCACC |
| mSox2-qR | TGGAGTTGTACTGCAGGGCG |
| mKlf4-qF | GCACACCTGCGAACTCACAC |
| mKlf4-qR | CCGTCCCAGTCACAGTGGTAA |
| mKlf2-qF | CTCAGCGAGCCTATCTTGCC |
| mKlf2-qR | CACGTTGTTTAGGTCCTCATCC |
| mTfcp2l1-qF | CAGCCCGAACACTACAACCAG |
| mTfcp2l1-qR | CAGCCGGATTTCATACGACTG |
| mTfap2c-qF | ATCCCTCACCTCTCCTCTCC |
| mTfap2c-qR | CCAGATGCGAGTAATGGTCGG |
| mNestin-qF | CCCTGAAGTCGAGGAGCTG |
| mNestin-qR | CTGCTGCACCTCTAAGCGA |
| mTuj1-qF | CCCAGCGGCAACTATGTAGG |
| mTuj1-qR | CCAGACCGAACACTGTCCA |
